# Identification of genetic loci and candidate genes regulating photosynthesis and leaf morphology through genome-wide association study in *Brassica napus* L.

**DOI:** 10.3389/fpls.2024.1467927

**Published:** 2024-12-20

**Authors:** Keqi Li, Na Guo, Miao Zhang, Yuanyuan Du, Jiali Xu, Shimeng Li, Jinxiong Wang, Rongrong Wang, Xiang Liu, Mengfan Qin, Yu Xu, Yunlin Zhu, Jia Song, Aixia Xu, Zhen Huang

**Affiliations:** ^1^ State Key Laboratory of Crop Stress Biology for Arid Areas/College of Agronomy, Northwest A&F University, Yangling, Shaanxi, China; ^2^ College of Life Sciences, Shanxi Agricultural University, Taiyuan, Shanxi, China; ^3^ Agricultural Research Institute of Tibet Autonomous Region Agriculture and Animal Husbandry Sciences, Lhasa, Tibet, China

**Keywords:** rapeseed, photosynthesis, leaf morphology, GWAS, QTN, candidate genes

## Abstract

Rapeseed (*Brassica napus* L.) is a major agricultural crop with diverse applications, particularly in the production of seed oil for both culinary use and biodiesel. However, its photosynthetic efficiency, a pivotal determinant of yield, remains relatively low compared with other C_3_ plants such as rice and soybean, highlighting the necessity of identifying the genetic loci and genes regulating photosynthesis in rapeseed. In this study, we investigated 5 photosynthesis traits and 5 leaf morphology traits in a natural population of rapeseed, and conducted a genome-wide association study (GWAS) to identify significantly associated loci and genes. The results showed that the gas-exchange parameters of the dark reactions in photosynthesis exhibited a significant positive correlation with the chlorophyll content, whereas they showed a weaker negative correlation with the leaf area. By GWAS, a total of 538 quantitative trait nucleotides (QTNs) were identified as significantly associated with traits related to both leaf morphology and photosynthesis. These QTNs were classified into 84 QTL clusters, of which, 21 clusters exhibited remarkable stability across different traits and environmental conditions. In addition, a total of 3,129 potential candidate genes were identified to be significantly associated with the above-mentioned 10 traits, most of which were shared by certain traits, further indicating the reliability of the findings. By integrating GWAS data with GO enrichment analysis and gene expression analysis, we further identified 8 key candidate genes that are associated with the regulation of photosynthesis, chlorophyll content, leaf area, and leaf petiole angle. Taken together, this study identified key genetic loci and candidate genes with the potential to improve photosynthetic efficiency in rapeseed. These findings provide a theoretical framework for breeding new rapeseed varieties with enhanced photosynthetic capabilities.

## Introduction

Rapeseed (*Brassica napus* L.) is extensively cultivated worldwide and serves as a versatile crop with applications ranging from seed oil production to biodiesel (Li J. et al., 2021; Raboanatahiry et al., 2021). Additionally, rapeseed contributes to agriculture through its use as fodder, ornamental plants, vegetables, and organic fertilizer, thereby exhibiting great significance in both the agricultural economy and environmental sustainability.

Photosynthesis plays a vital role in crop yield by providing the energy and carbohydrates necessary for both vegetative and reproductive growth of plants. The efficiency of light energy conversion determines crop yield. Despite its agricultural importance, rapeseed, as a C_3_ plant, exhibits comparatively lower light energy utilization efficiency than C_4_ plants like maize and sorghum (Wang et al., 2014), and even other C_3_ crops such as rice (Honda et al., 2023) and soybean (Wang et al., 2020). Leaves are the primary organs responsible for photosynthesis in rapeseed, and during the reproductive growth phase, their photosynthetic capacity has a profound impact on the yield and quality of seed oil. The production and accumulation of dry matter within the plant during this stage are closely linked to the silique number per plant and the seed number per silique (Allen and Morgan, 1972; Ramana and Ghildiyal, 2010). Therefore, improving the light energy utilization rate of rapeseed is one of the key strategies to increase its yield.

Photosynthetic efficiency of plants is governed by complex biological processes, and influenced by multiple factors, mainly including light intensity, CO_2_ concentration, temperature, water availability, chlorophyll content, stomatal opening, enzyme activity, plant species, nutritional status, and environmental conditions. For example, overexpression of rubisco enzyme in the Calvin cycle has been reported to increase the regeneration of ribulose-1,5-bisphosphate (*RuBP*), thus improving the photosynthetic efficiency in multiple C3 plants such as Arabidopsis (Simkin et al., 2017), tobacco (Miyagawa et al., 2001), potato (Vijayakumar et al., 2024), and soybean (Köhler et al., 2017). Similarly, overexpression of the chloroplast *NAD kinase* (*NADK2*) significantly promotes photosynthetic electron transport, thereby improving photosynthesis (Takahara et al., 2010). In recent years, studies have been conducted to explore high photosynthetic efficiency germplasm in various crops, aiming to enhance photosynthetic efficiency either directly or indirectly. Some studies have focused on identifying genes or loci that can increase the chlorophyll content, thus indirectly enhancing photosynthetic efficiency (Gudi et al., 2023; Jin et al., 2023; Lin et al., 2022; Ye et al., 2020), while other studies have employed genome-wide association studies (GWAS) to directly identify genes or loci influencing photosynthetic traits in natural populations. GWAS has been widely used for analyzing the genetic basis of various complex traits in rapeseed (Han et al., 2022; Hu et al., 2023; Li K. et al., 2021; Wang et al., 2022; Zhang et al., 2024), and has also been applied to pinpoint genetic regions related to different chlorophyll fluorescence characteristics in many other crops, including rice (Hamdani et al., 2019), barley (Rapacz et al., 2019), soybean (Herritt et al., 2018), maize (Jin et al., 2023), and Arabidopsis (Rungrat et al., 2019). However, GWAS has been less frequently employed to investigate gas-exchange parameters of dark reactions in photosynthesis (such as CO_2_ fixation dynamics) in crops like maize, rice, wheat, and soybean (Hu et al., 2024; Shamim et al., 2022; Wang et al., 2020). To date, few reports have been conducted on the identification of genes related to photosynthetic efficiency from rapeseed, likely due to challenges in accurately measuring these traits and identifying relevant loci. Identifying genes that enhance photosynthetic efficiency will provide valuable targets for breeding programs aimed at developing improved rapeseed varieties.

Photosynthesis predominantly occurs within chloroplasts, which are predominantly found in leaf cells. Chlorophyll within these chloroplasts plays a crucial role in capturing sunlight and converting it into chemical energy. As the main photosynthetic organs, leaves exhibit morphological traits—such as leaf length (LL), leaf width (LW), leaf area (LA), leaf petiole angle (PA), and petiole length (PL)—that can influence overall photosynthetic performance.

Therefore, this study first identified the photosynthesis traits of rapeseed leaves and the leaf morphology traits across different germplasms, followed by an analysis of the genetic relationships between these traits. Finally, we identified loci and genes associated with leaf morphology and photosynthetic rate using GWAS. Our findings provide a theoretical foundation and technical framework for breeding novel rapeseed varieties with enhanced photosynthetic efficiency, yield, and oil content.

## Materials and methods

### Plant materials and field management

A highly diverse natural population comprising 104 core inbred lines of rapeseed were sourced from the germplasm resource laboratory at Northwest Agriculture and Forestry University (NWAFU, Shaanxi province, China) (Qin et al., 2022). These lines were cultivated and evaluated at the Caoxinzuang experimental station of the NWAFU, Yangling, Shaanxi province (latitude 34°30’ N, longitude 108°9’ E), across 3 continuous cropping seasons (2018-2019, 2019 - 2020, and 2020-2021). A randomized block experimental design was employed, with each line planted in 3 rows with a row spacing of 0.35 m × 0.15 m and a row length of 2 m. Sowing was carried out in September of the previous year, and the routine field management was implemented.

### Phenotype evaluation

At the 8- to 10-leaf stage, we assessed 5 leaf morphology traits, including leaf length (LL), leaf width (LW), leaf area (LA), petiole angle (PA), and petiole length (PL) and 5 photosynthesis-related traits, including the net photosynthetic rate (Pn), stomatal conductance (Gs), intercellular carbondioxide concentration (Ci), the transpiration rate (Tr), and leaf chlorophyll content (Chl). The leaf chlorophyll content was measured during the seedling stage using a SPAD-502 meter. In the seedling phase, the fifth fully expanded leaf was selected for photosynthetic trait assessment, and the assessment was performed utilizing the LI-6400 portable photosynthesis system (LI-COR 6400XT, LI-COR, Lincoln, NE, USA) between 9:00 and 11:00 am on sunny mornings. Light intensity was set at 500 μmol m^-2^s^-1^, relative humidity of the air was maintained at 40%, and the CO_2_ concentration was adjusted to 450 μmol mol^-1^. Each experiment included three biological replicates, and five plants per line were randomly selected as sample replicates. Each plant was measured three times for technical replication to ensure accuracy, and the average value for each line was calculated based on these five plants.

### Data analyses

Phenotypic data were analyzed using Excel and R 4.2.1 software. The best linear unbiased prediction (BLUP) value for each inbred line was obtained by fitting a mixed linear model using the R/lme4 package. Heritability (h^2^) was calculated according to the following formula (Knapp, 1986):


(1) $${\text{h}}^{2}=\sigma_{\text{G}}^{2}/\left(\sigma_{\text{GE}}^{2}/\text{n}+\sigma_{\text{G}}^{2}+\sigma_{\text{e}}^{2}/\text{nr}\right)$$

where, genotypic variance ( $\sigma_{\text{G}}^{2}$ ), genotype × environment variance ( $\sigma_{\text{GE}}^{2}$ ), and error variance ( $\sigma_{\text{e}}^{2}$ ) were estimated based on the number of years (n) and replicates (r). These estimates were analyzed using the lmer function in the R/lme4 package. Correlation and partial correlation analyses among the traits were conducted using PerformanceAnalytics v.2.0.4 in the R package (Peterson et al., 2014).

### GWAS of leaf morphology, chlorophyll content, and photosynthesis-related traits

The Brassica 50K Illumina Infinium consortium SNP array was employed for genotyping (Qin et al., 2022). The SNPs were screened using the TASSEL5 software, with the following thresholds: a missing rate ≤ 0.2, a heterozygous rate ≤ 0.2, and a minor allele frequency (MAF) > 0.05 (Bradbury et al., 2007). As a result, a total of 22,628 SNPs were selected for GWAS analysis. The genetic structure and kinship relationships of the 104 germplasms were examined using the STRUCTURE v. 2.3.4 software and SPAGeDi, respectively. Based on annual data and the BLUP values, associations between the traits (leaf morphology, chlorophyll content, and photosynthesis-related traits) and SNPs were investigated using a mixed linear model (MLM) in the TASSEL5 software (Bradbury et al., 2007). SNPs with -log_10_ p-value > 4 were defined as significantly associated with the phenotype. Haplotypes at the loci associated with traits were identified using the four-gamete rule through the Haploview software (Wei et al., 2016). The R 4.2.1 software was used for the generation of box plots and the visualization of the relative phenotypic data.

### Candidate gene identification

Candidate loci were defined as those detected across at least two traits or consistently identified across multiple years (2019, 2020, and 2021). The *B. napus* reference genome Darmor-bzh v5 (https://www.genoscope.cns.fr/brassicanapus) was used to identify candidate genes (Chalhoub et al., 2014). Genes located within linkage disequilibrium (LD) blocks (r^2^ > 0.6) of significant SNPs were defined as potential candidate genes (Dong et al., 2021). Additionally, the genes outside LD blocks but within a 100 Kb flanking region were also designated as potential candidate genes (Khanzada et al., 2020; Zhou et al., 2018). Subsequently, the potential candidate genes were subjected to functional annotations through protein BLAST searches in NCBI (https://www.ncbi.nlm.nih.gov/). The homologous genes of these candidate genes were identified from Arabidopsis and rapeseed cultivar ‘ZS11’ reference genomes in TAIR (https://www.arabidopsis.org) and BnIR (https://yanglab.hzau.edu.cn) databases by BLAST analysis, respectively. GO (Gene ontology) enrichment analysis of these potential candidate genes was conducted utilizing the ClusterProfiler 4.0 package in R (Wu et al., 2021). Subsequently, gene expression profiles across five developmental tissues (root, leaf, stem, bud, and silique) were obtained from BnIR (Yang et al., 2023).

## Results

### Phenotypic variations

**Figure 1:**
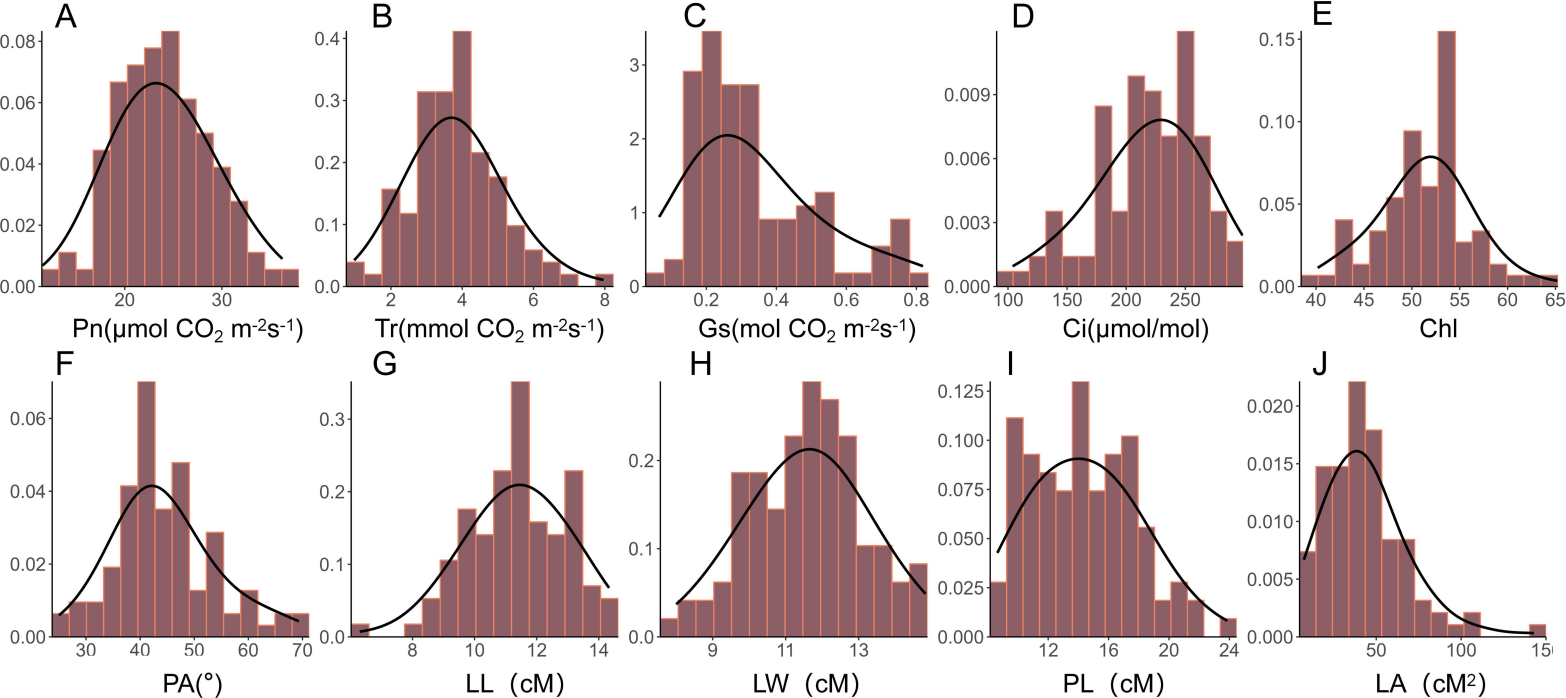
Histogram of the frequency distribution of photosynthetic and leaf morphological traits. **(A-J)** Frequency distribution histograms of net photosynthetic rate (Pn), transpiration rate (Tr), stomatal conductance (Gs), intercellular carbondioxide concentration (Ci), chlorophyll content of leaf (Chl), petiole angle (PA), leaf length (LL), leaf width (LW), petiole length (PL), and leaf area (LA) in the natural population of rapeseed.

Photosynthesis is a complex process influenced by various environmental factors throughout plant growth, and thus cannot be adequately characterized by a single parameter. In this study, a total of 10 leaf morphology- and photosynthesis- related traits were evaluated, including leaf length (LL), leaf width (LW), leaf area (LA), petiole angle (PA), petiole length (PL), leaf chlorophyll content (Chl), net photosynthetic rate (Pn), intercellular carbondioxide concentration (Ci), stomatal conductance (Gs), and transpiration rate (Tr) of a nature population of rapeseed. The frequency distribution histogram demonstrated that most of these traits followed a normal distribution or were approximately normal, except for LA and Gs, which exhibited left skewness (**Figure 1**). Significant phenotypic variation was observed across all traits under different environmental conditions, with coefficients of variation (CV) ranging from 7.92% to 53.22% (**Table 1**). Among the 10 traits, Chl content exhibited the lowest variation, with a CV value of 9.14%, 9.33%, and 9.70% in 2019, 2020, and 2021, respectively. Conversely, Gs displayed the highest variation, with a CV value of 52.30%, 53.22%, and 39.36% in 2019, 2020, and 2021, respectively. These findings indicate that the materials used in our study exhibited substantial diversity in photosynthesis-related traits. Moreover, the broad-sense heritability (H^2^) of these 10 traits was computed in all three years (2019, 2020, and 2021), revealing moderate to high heritability values, ranging from 62% for Tr to 74% for Ci (**Table 1**). These results suggest that the genetic factors were the primary contributors to the leaf photosynthesis-related phenotypic variations, thereby making these traits suitable for subsequent GWAS analysis.

**Table 1:** Variation of five leaf morphology traits and five leaf photosynthesis traits.

Traits	Environment	Min	Max	Mean	SD	CV(%)	H2	H2
LL(cM)	BLUP	6.51	12.77	9.47	1.12	11.78	–	0.71
	2019	6.30	14.33	11.39	1.54	13.54	0.79	
	2020	4.83	11.70	8.14	1.19	14.58	0.78	
	2021	4.63	13.23	9.64	1.44	14.91	0.83	
LW(cM)	BLUP	6.96	13.80	9.79	1.06	10.79	–	0.75
	2019	8.00	14.83	11.57	1.52	13.14	0.85	
	2020	4.27	12.40	8.44	1.17	13.84	0.81	
	2021	6.67	15.70	11.15	1.43	12.79	0.80	
PL(cM)	BLUP	8.59	21.33	12.46	2.20	17.62	–	0.65
	2019	8.63	23.87	14.38	3.39	23.55	0.88	
	2020	6.03	15.87	10.92	2.02	18.47	0.78	
	2021	8.17	19.90	14.09	2.81	19.96	0.75	
PA(°)	BLUP	16.25	50.00	32.57	6.13	18.81	–	0.61
	2019	25.00	69.33	44.47	9.21	20.72	0.89	
	2020	11.33	42.67	26.36	5.94	22.55	0.80	
	2021	8.33	50.00	25.28	7.67	30.32	0.86	
LA(cM^2^)	BLUP	37.55	127.70	87.33	18.70	21.41	–	0.68
	2019	7.35	142.88	43.58	23.87	54.77	0.84	
	2020	36.17	149.83	83.14	20.42	24.56	0.91	
	2021	52.70	194.75	122.07	28.78	23.58	0.93	
Pn(μmol CO_2_ m^-2^s^-1^)	BLUP	7.34	23.09	15.95	2.98	18.66	–	0.64
	2019	11.53	36.23	23.92	4.86	20.30	0.72	
	2020	3.15	21.30	12.64	3.74	29.56	0.81	
	2021	10.43	26.75	18.32	2.99	16.32	0.80	
Gs(mol CO_2_ m^-2^s^-1^)	BLUP	0.08	0.50	0.22	0.09	40.18	–	0.66
	2019	0.07	0.82	0.34	0.18	52.30	0.86	
	2020	0.03	0.46	0.18	0.09	53.22	0.85	
	2021	0.11	0.60	0.26	0.10	39.36	0.81	
Ci(μmol/mol)	BLUP	153.09	298.56	231.62	26.55	11.46	–	0.74
	2019	103.89	298.56	218.30	42.51	19.47	0.86	
	2020	161.52	310.69	239.84	28.97	12.08	0.80	
	2021	148.17	298.56	229.36	27.89	12.16	0.84	
Tr(mmol CO_2_ m^-2^s^-1^)	BLUP	1.44	4.89	2.91	0.72	24.66	–	0.62
	2019	0.97	7.96	3.82	1.26	32.92	0.86	
	2020	0.68	4.64	2.38	0.92	38.48	0.85	
	2021	1.74	5.55	3.11	0.75	24.23	0.85	
Chl(SPAD)	BLUP	39.79	57.45	47.69	3.78	7.92	–	0.67
	2019	40.17	65.20	51.32	4.69	9.14	0.75	
	2020	31.57	57.13	43.71	4.08	9.33	0.72	
	2021	37.23	61.67	48.19	4.67	9.70	0.93	

### Correlation analyses between leaf morphology traits and photosynthesis traits

**Figure 2:**
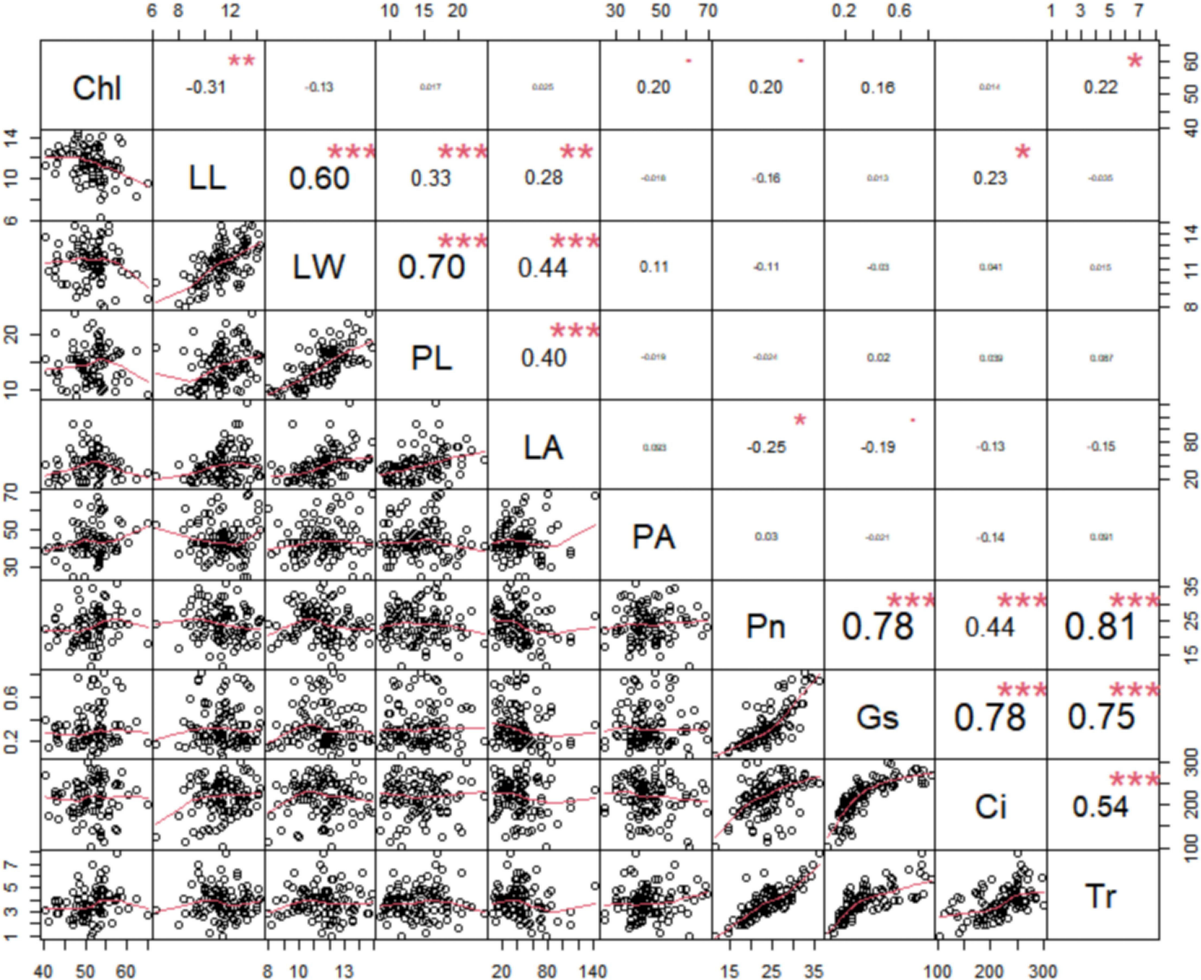
Pairwise Pearson correlation among the ten photosynthetic and leaf morphological traits in rapeseed. The upper triangle of the matrix illustrates the Pearson correlation coefficients for each pair of traits, with positive values indicating positive correlations and negative values indicating inverse correlations. Conversely, the lower triangle displays the linear regression statistics for pairs of traits. net photosynthetic rate (Pn), leaf chlorophyll content (Chl), transpiration rate (Tr), intercellular carbondioxide concentration (Ci), stomatal conductance (Gs), petiole angle (PA), leaf area (LA), leaf length (LL), leaf width (LW), and petiole length (PL). ***: Indicates p-value less than 0.001, **: Indicates p-value less than 0.01, *: Indicates p-value less than 0.05, The font size represents the strength of the correlation, with larger fonts indicating stronger correlations and smaller fonts indicating weaker correlations.

Pearson correlation analysis was performed to uncover the correlation between the leaf morphology traits and photosynthesis traits. The results showed that leaf area was positively correlated with leaf length, leaf width, and petiole length, while displaying a weaker negative correlation with the photosynthetic rate and stomatal conductance (**Figure 2**). Additionally, all 5 photosynthesis traits were positively correlated with each other, with the Chl content showing a positive correlation with petiole angle (**Figure 2**). These findings suggest that while leaf morphology does not directly affect intrinsic photosynthetic capacity, it may influence light penetrance.

### Genome-wide association analysis for photosynthesis and leaf morphology traits

**Figure 3:**
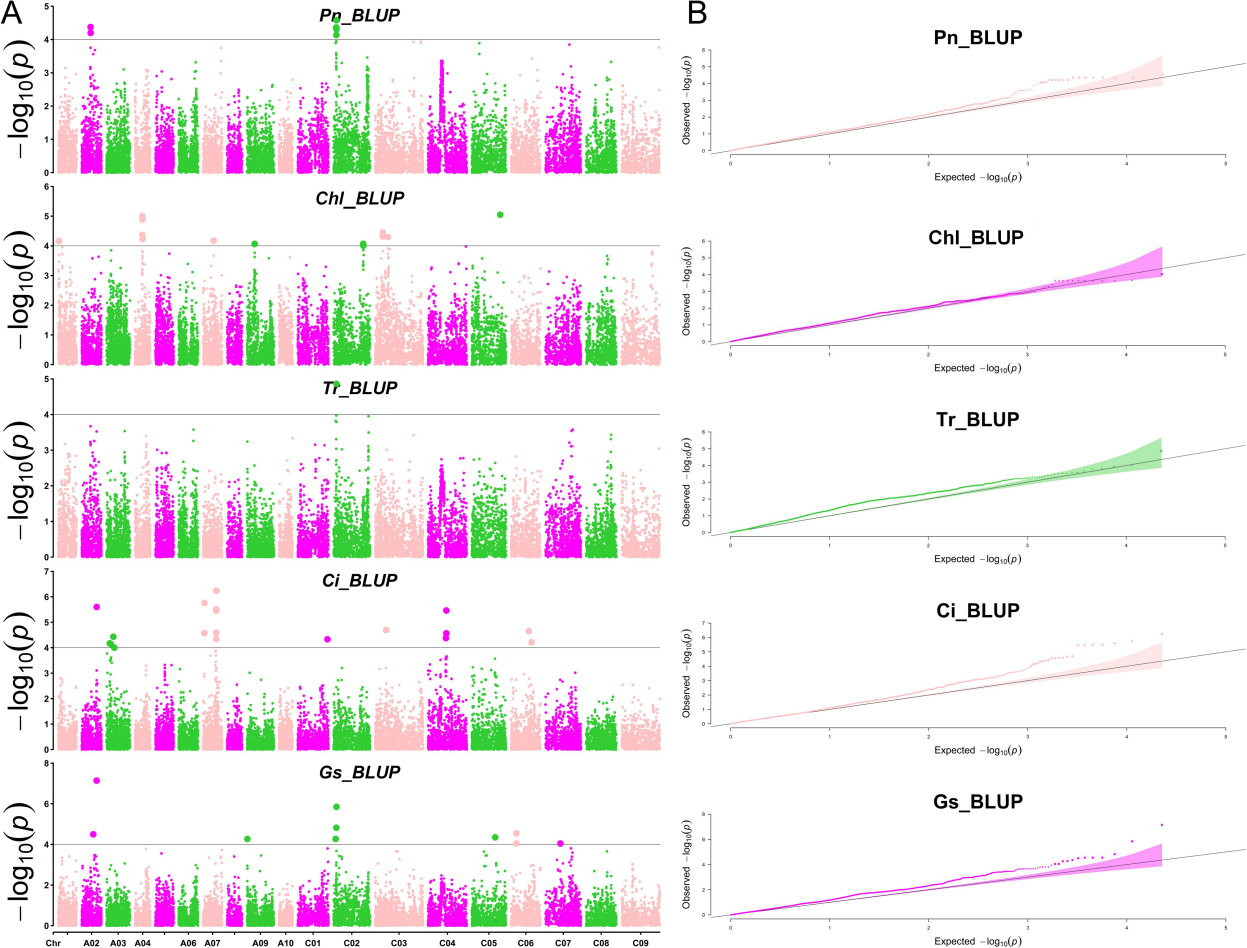
Manhattan and QQ plots of photosynthesis-related traits by GWAS. **(A)** The Manhattan plots of net photosynthetic rate (Pn), leaf chlorophyll content (Chl), transpiration rate (Tr), intercellular carbondioxide concentration (Ci), and stomatal conductance (Gs) by GWAS (from top to bottom). **(B)** The QQ plots of net photosynthetic rate (Pn), leaf chlorophyll content (Chl), transpiration rate (Tr), intercellular carbondioxide concentration (Ci), and stomatal conductance (Gs) by GWAS (from top to bottom).

**Figure 4:**
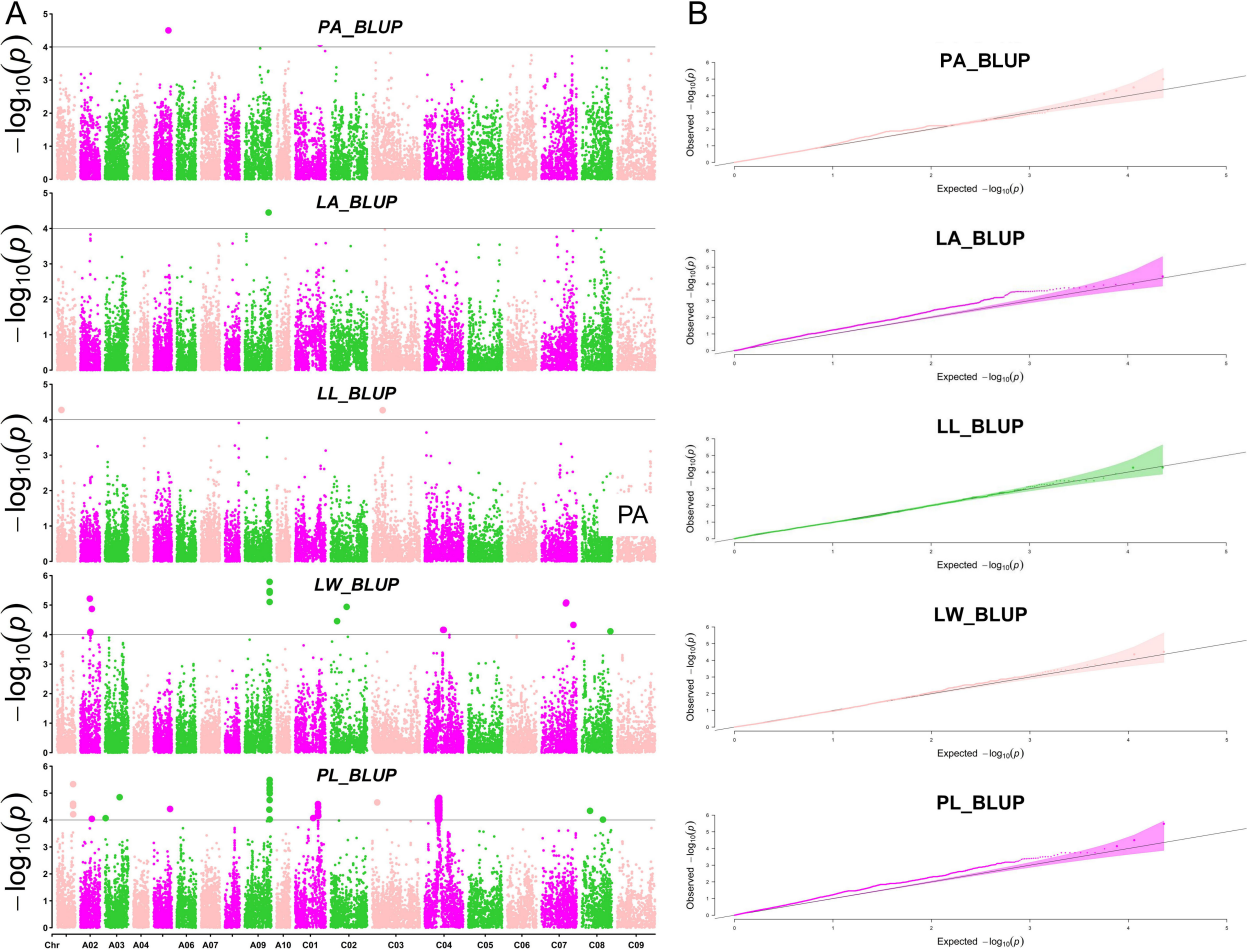
Manhattan and QQ plots of leaf morphology-related traits by GWAS. **(A)** The Manhattan plots of petiole angle (PA), leaf area (LA), leaf length (LL), leaf width (LW), and petiole length (PL) (from top to bottom). **(B)** The QQ plots of petiole angle (PA), leaf area (LA), leaf length (LL), leaf width (LW), and petiole length (PL) (from top to bottom).

**Figure 5:**
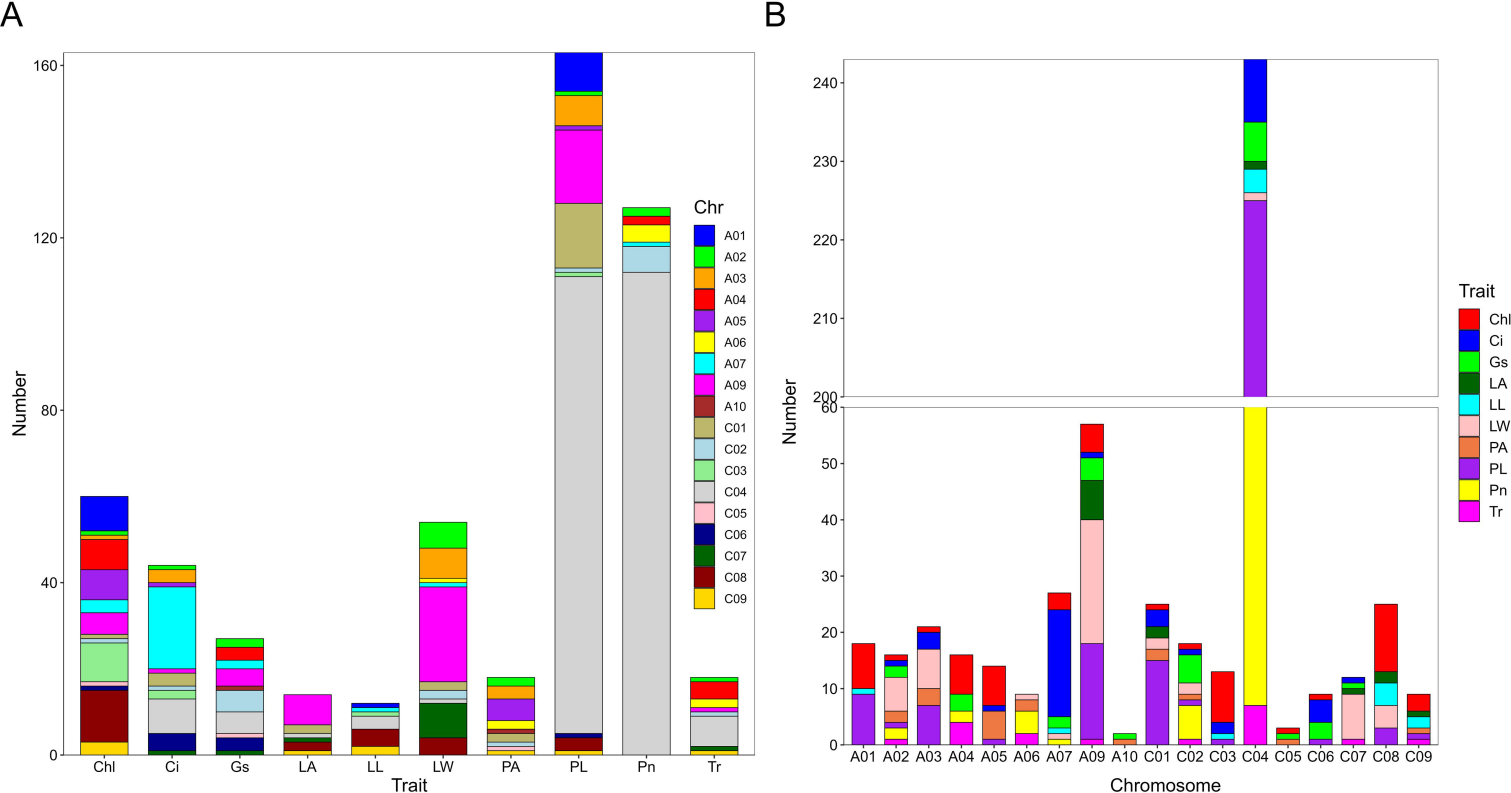
Number of QTNs associated with photosynthetic and leaf morphological traits. **(A)** Number of QTNs significantly associated with each trait. The horizontal axis represents different traits, and the vertical axis represents the number of detected QTNs. Different colors represent QTNs identified on different chromosomes. **(B)** Number of QTNs significantly associated with traits on different chromosomes. The horizontal axis represents different chromosomes, and the vertical axis represents the number of detected QTNs. Different colors represents QTNs identified by different traits. net photosynthetic rate (Pn), leaf chlorophyll content (Chl), transpiration rate (Tr), intercellular carbondioxide concentration (Ci), stomatal conductance (Gs), petiole angle (PA), leaf area (LA), leaf length (LL), leaf width (LW), and petiole length (PL).

To identify loci significantly associated with leaf morphology and photosynthesis, GWAS was conducted using three years of data along with BLUP values. **Figures 3** and **4** illustrate the Manhattan plots derived from the GWAS based on BLUP values for photosynthesis and leaf traits, respectively. The GWAS results showed that a total of 538 quantitative trait nucleotides (QTNs) were identified to be significantly associated with leaf area and photosynthesis traits (−log_10_^P >^4) across the three years (**Supplementary Table S1**), of which, 122 QTNs were found to be associated with multiple traits in different years (**Supplementary Table S2**). The largest number (163) of significant QTNs were identified to be associated with PL, followed by 127, 60, 54, 44, 27, 18, 18, 14, and 12 QTNs with Pn, Chl, LW, Ci, Gs, Tr, PA, LA, and, LL, respectively (**Figure 5A**).These 538 significant QTNs were distributed across all chromosomes except A08, with the highest number on chromosome C04, followed by chromosome A09 (**Figure 5B**). QTNs located in close proximity (< 1 Mb) and in linkage disequilibrium (LD) (r^2^> 0.2) were grouped into the same cluster, since they correspond to the same quantitative trait locus (QTL) (Liu et al., 2016). Therefore, these 538 QTNs were categorized into 84 QTL clusters (**Supplementary Table S1**).

We further analyzed the QTNs co-detected to be associated with different traits and planting years. Among the total 84 QTL clusters, 21 were identified as key clusters, as they were associated with two or more traits or consistently associated with the same traits across multiple planting years (**Supplementary Table S3**). These 21 key QTL clusters contained 381 QTNs, which were distributed on 12 chromosomes (including A02, A03, A04, A05, A07, A08, A09, C01, C02, C04, C06, and C07) (**Supplementary Figure S1**). Among these 21 key QTL clusters, 8, 6, 6, 5, 5, 4, 3, 3, 2, and 2 clusters were associated with LW, Gs, PL, Ci, Tr, Pn, Chl, PA, LA, and LL, respectively (**Table 2**). Cluster *q.A3-5* was co-identified to be associated with Ci, LW, and PL traits, explaining 15.96%~23.42% of the phenotypic variation. Cluster *q.A4-2* and *q.C2-2* were co-identified to be associated with Gs, Tr, and Pn traits, explaining 17.85%~22.35% and 23.48%~30.18% of the phenotypic variation, respectively. Cluster *q.A7-4* was co-identified to be associated with Gs, Pn, LW, and LL traits, explaining 18.29%~22.05% of the phenotypic variation. Cluster *q.A9-1* was associated with Gs, Ci, and Pn traits, explaining 18.11%~27.2% of the phenotypic variation. Cluster *q.A9-4* was associated with PL, LW, and LA, explaining 16.18%~41.62% of the phenotypic variation. The *q.C4-2* was associated with Gs, Tr, Pn, LL, and PL, explaining 14.7%~20.66% of the phenotypic variation. The *q.C7-2* was associated with Gs, Ci, Tr, and LW, explaining 15.83%~22.26% of the phenotypic variation (**Table 2**; **Supplementary Figure S1**). These QTLs, detected across multiple traits, are considered relatively stable and thus present valuable targets for improving photosynthetic efficiency in rapeseed.

**Table 2:** Key QTL clusters identified by GWAS.

Trait	QTL Cluster	Peak SNP	Chr	Pos	−log_10_(P)	PVE(%)	Haplotype Block (Mb)
Chl	*q.A4-1*	A04_9347569	A04	9347569	5.37	23.57	8.937-9.891
	*q.A9-2*	A09_8460857	A09	8460857	4.25	18.80	8.230-10.068
	*q.C1-6*	C01_29895643	C01	29895643	4.22	17.70	25.894-31.792
Pn	*q.A4-2*	A04_15190622	A04	15190622	5.11	21.55	14.374-15.334
	*q.A7-4*	A07_22302428	A07	22302428	4.26	18.29	22.297-24.003
	*q.C2-2*	C02_3694393	C02	3694393	5.85	30.18	3.163-3.797
	*q.C4-2*	C04_16641010	C04	16641010	4.86	20.60	14.272-19.112
Tr	*q.A2-6*	A02_18822332	A02	18822332	4.93	22.74	18.780-19.312
	*q.A4-2*	A04_15195933	A04	15195933	4.74	20.14	14.374-15.334
	*q.A9-1*	A09_325988	A09	325988	5.24	21.84	0.034-0.059
	*q.C4-2*	C04_16928385	C04	16928385	4.26	15.52	14.272-19.112
	*q.C7-2*	C07_32755413	C07	32755413	4.65	19.63	30.599-32.365
Ci	*q.A2-6*	A02_18822332	A02	18822332	5.60	24.78	18.780-19.312
	*q.A3-5*	A03_5625219	A03	5625219	4.14	15.96	5.59-6.239
	*q.A7-2*	A07_16847439	A07	16847439	6.24	27.20	15.273-17.063
	*q.A9-1*	A09_325988	A09	325988	4.30	18.11	0.034-0.59
	*q.C7-2*	C07_32755413	C07	32755413	4.07	15.83	30.599-32.365
Gs	*q.A2-6*	A02_18822332	A02	18822332	7.14	30.90	18.780-19.312
	*q.A4-2*	A04_15190622	A04	15190622	5.33	22.35	14.374-15.334
	*q.A7-4*	A07_22302428	A07	22302428	5.16	21.71	22.297-24.003
	*q.A9-1*	A09_325988	A09	325988	6.76	27.20	0.034-0.59
	*q.C4-2*	C04_16928385	C04	16928385	4.39	15.99	14.272-19.112
	*q.C7-2*	C07_32755413	C07	32755413	4.57	19.32	30.599-32.365
PA	*q.A3-8*	A03_19067727	A03	19067727	4.41	18.71	18.420-19.083
	*q.A5-4*	A05_18835949	A05	18835949	5.73	24.47	18.730-19.028
	*q.C1-6*	C01_31701754	C01	31701754	5.96	33.21	25.894-31.792
LA	*q.A9-3*	A09_29345053	A09	29345053	4.90	21.16	29.089-29.71
	*q.A9-4*	A09_31800469	A09	31800469	4.57	17.69	30.333-31.813
LW	*q.A2-5*	A02_14502746	A02	14502746	4.87	20.27	14.100-14.601
	*q.A2-7*	A02_23303646	A02	23303646	4.95	21.55	23.175-23.644
	*q.A3-5*	A03_6179649	A03	6179649	5.56	23.42	5.59-6.239
	*q.A7-4*	A07_23885182	A07	23885182	4.33	21.71	22.297-24.003
	*q.A9-3*	A09_29345053	A09	29345053	6.97	28.43	29.089-29.71
	*q.A9-4*	A09_31652307	A09	31652307	11.70	40.76	30.333-31.813
	*q.C1-6*	C01_30553786	C01	30553786	4.42	17.10	25.894-31.792
	*q.C7-2*	C07_31419060	C07	31419060	5.09	22.26	30.599-32.365
LL	*q.A7-4*	A07_23539032	A07	23539032	4.54	19.39	22.297-24.003
	*q.C4-2*	C04_15255069	C04	15255069	4.13	17.98	14.272-19.112
PL	*q.A2-7*	A02_23303646	A02	23303646	4.46	19.65	23.175-23.644
	*q.A3-5*	A03_5681359	A03	5681359	4.41	19.42	5.59-6.239
	*q.A9-4*	A09_31652307	A09	31652307	8.19	29.98	30.333-31.813
	*q.C1-6*	C01_28998632	C01	28998632	4.59	19.78	25.894-31.792
	*q.A3-8*	A03_18670129	A03	18670129	4.85	20.75	18.420-19.083
	*q.C4-2*	C04_16601979	C04	16601979	4.72	20.06	14.272-19.112

### Allelic effects of stable QTNs on photosynthesis and leaf morphology

**Figure 6:**
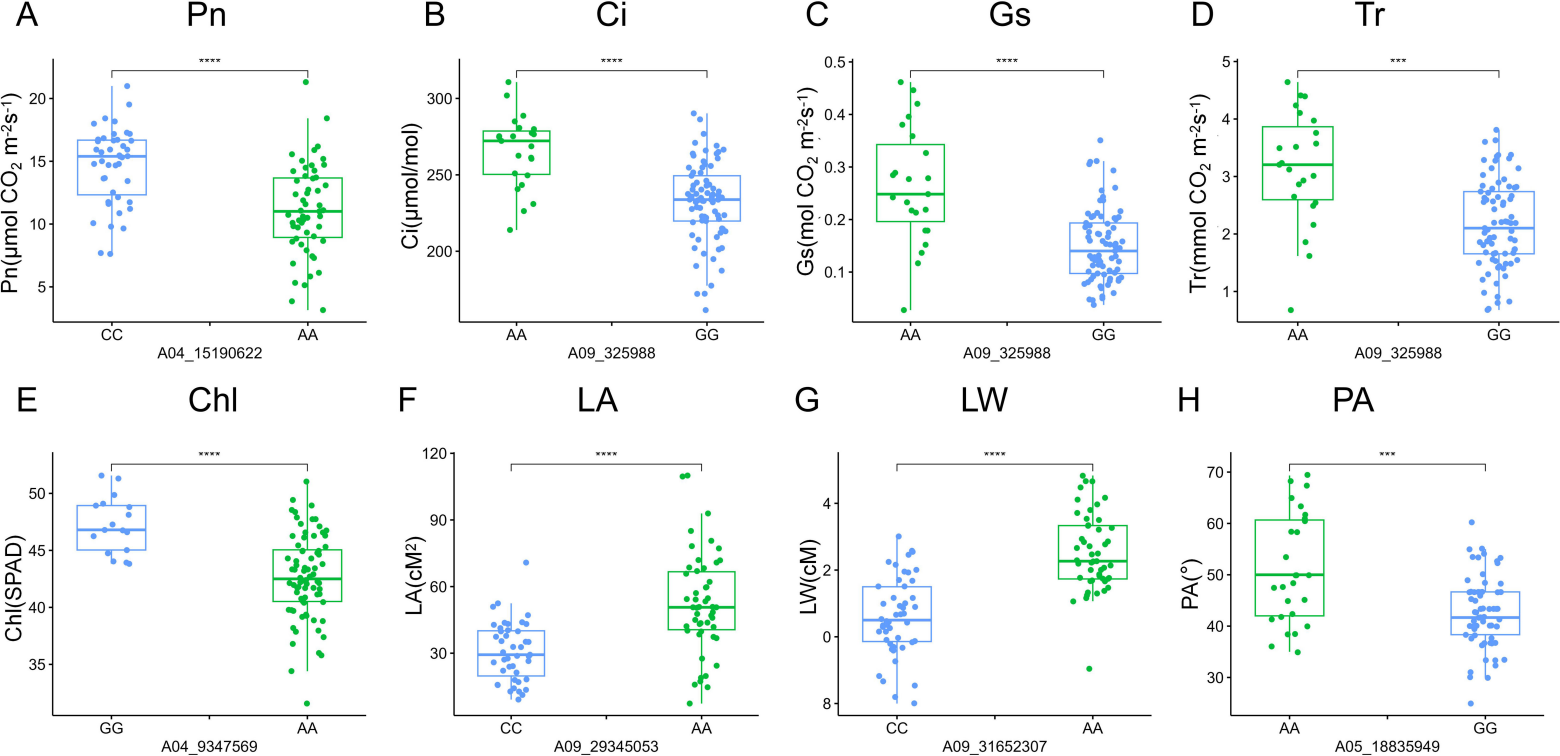
Phenotypic difference between two genotypes of QTNs significantly associated with different traits. **(A-H)** Box plots of allelic phenotypic variations of QTNs for the following traits: net photosynthetic rate (Pn), intercellular carbondioxide concentration (Ci), stomatal conductance (Gs), transpiration rate (Tr), leaf chlorophyll content (Chl), leaf area (LA), leaf width (LW), and petiole angle (PA). The vertical axes of A-H represent Pn, Ci, Gs, Tr, Chl, LA, LW, and, PA, respectively. Significant differences between the alleles were assessed using a two-tailed t-test, *** indicates P < 0.001, **** indicates P < 0.0001.

To identify the beneficial alleles enhancing plant productivity in rapeseed, we compared the phenotypic differences between different alleles from these 21 QTL clusters (**Figure 6**; **Supplementary Figure S2**). The germplasms were divided into two subgroups based on their allele profiles, and the phenotypic values of these subgroups were analyzed. The results showed that rapeseed lines with the CC genotype at the locus A04_15190622 demonstrated a significantly higher net photosynthetic rate (Pn) compared with those with the AA genotype (**Figure 6A**). Moreover, the phenotypic values of intercellular carbondioxide concentration (Ci), stomatal conductance (Gs), transpiration rate (Tr), and leaf width (LW) of rapeseed plants with the CC genotype were also significantly higher than those with the AA genotype (**Supplementary Figure S2**), indicating that the locus A04_15190622 is a key QTN regulating the photosynthesis of rapeseed. The rapeseed plants with the AA genotype at the locus A09_325988 exhibited significantly higher phenotypic values of Ci, Gs, Tr, and LW compared with those with the GG genotype (**Figures 6B–D**; **Supplementary Figure S3**), indicating that the locus A09_325988 is also a key QTN influencing the photosynthesis process in rapeseed. The plants with the GG genotype at the locus A04_9347569 demonstrated a significantly higher Chl content compared with those with the AA genotype (**Figure 6E**), implying that the A04_9347569 is a crucial QTN that regulates the Chl content in rapeseed. The plants with the AA genotype at the locus A09_29345053 displayed a significantly larger leaf area (LA) and LW compared with those with the CC genotype (**Figures 6F, G**). Moreover, the phenotypic values of Tr, Chl, LL, and PL of the plants with the AA genotype were also significantly higher than those with the CC genotype (**Supplementary Figure S4**), suggesting that the locus A09_29345053 is a key QTN regulating leaf morphology in rapeseed. The plants carrying the AA genotype at the locus A05_18835949 exhibited a significantly larger petiole angle (PA) compared with those with the GG genotype (**Figure 6H**), indicating that the locus A05_18835949 is a crucial QTN regulating the petiole angle of rapeseed.

### Identification of candidate genes based on stable QTL clusters

**Figure 7:**
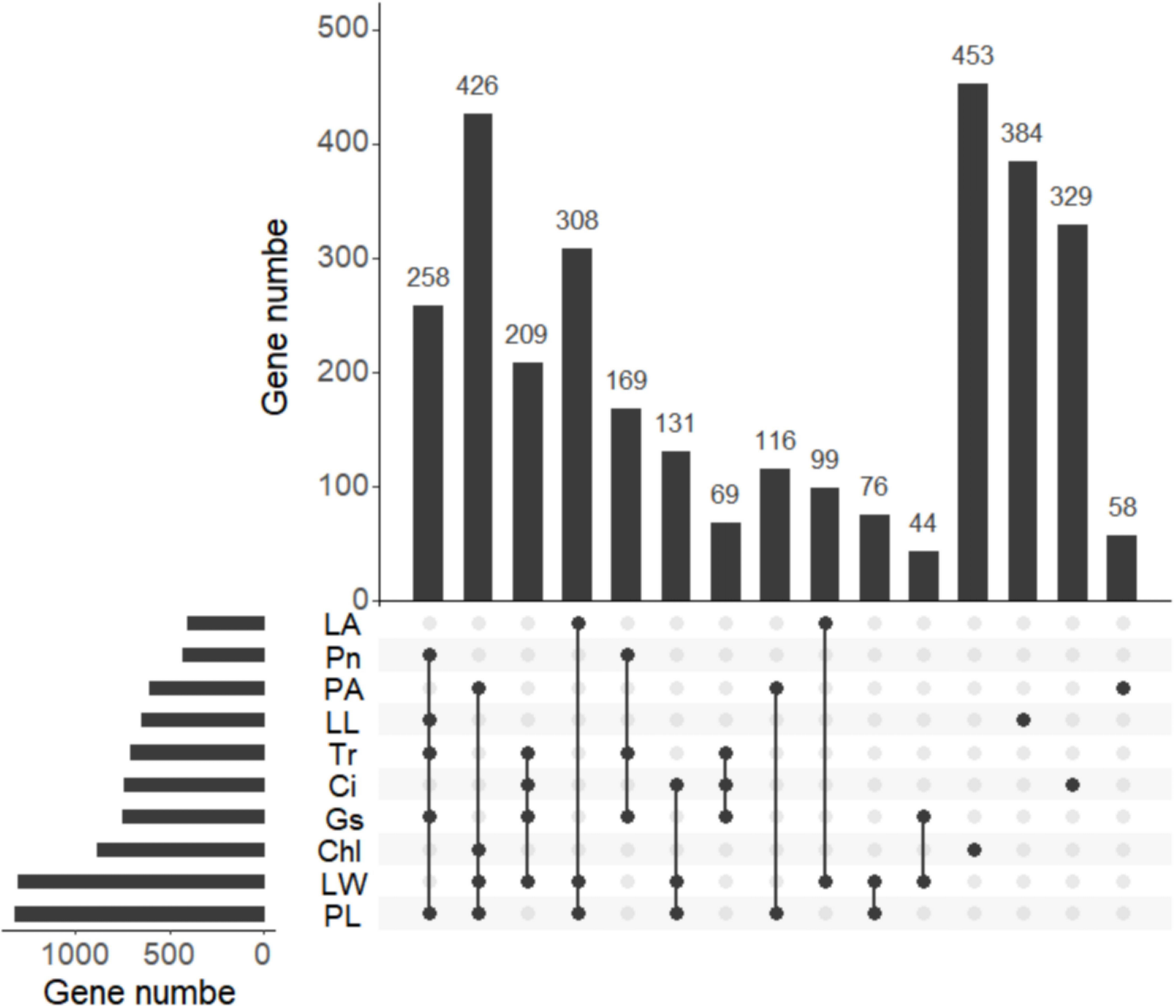
Venn of candidate genes detected for different traits. The histogram in the lower right corner shows the number of candidate genes associated with each trait. net photosynthetic rate (Pn), leaf chlorophyll content (Chl), transpiration rate (Tr), intercellular carbondioxide concentration (Ci), stomatal conductance (Gs), petiole angle (PA), leaf area (LA), leaf length (LL), leaf width (LW), and petiole length (PL).

**Figure 8:**
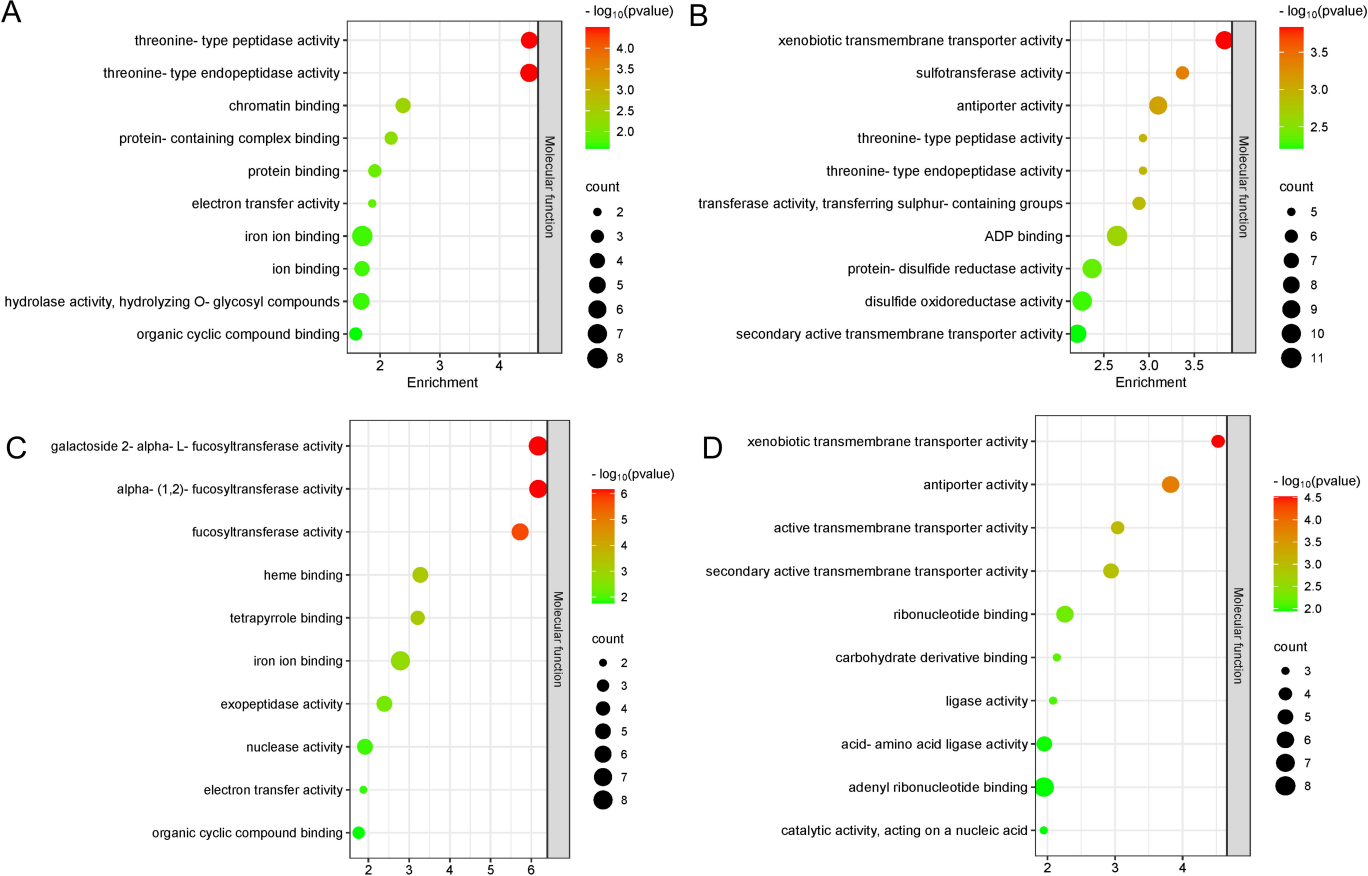
GO enrichment analysis of candidate genes. Top 10 GO terms significantly enriched with photosynthesis-related **(A)**, Chl content-related **(B)**, leaf area (LA)-related **(C)**, petiole angle (PA)-related **(D)** candidate genes.

A total of 3,129 potential candidate genes were identified from these 21 stable QTL clusters described above (**Table 2**). Among them, a total of 1,315 candidate genes were found in the QTL clusters for PL, followed by 1,293, 849, 749, 738, 705, 642, 600, 427, and 407 candidate genes for traits LW, Chl, Gs, Ci, Tr, LL, PA, Pn, and LA, respectively (**Figure 7**; **Supplementary Table S4**). A total of 258 candidate genes were shared by Pn, LL, Tr, Gs, and PL; 426 candidate genes by PA, Chl, LW, and PL; 209 candidate genes also by Ci, Tr, Gs, and LW; 308 candidate genes by LA, LW, and PL; 169 candidate genes by Pn, Tr, and Gs; 131 candidate genes by Ci, LW, and PL; 69 candidate genes by Ci, Tr, and Gs; 116 candidate genes by LL and PL; 99 candidate genes by LA and LW; 76 candidate genes by LW and PL; and 44 candidate genes by Gs and LW (**Figure 7**). These findings indicated that the candidate genes associated with different traits were overlapped, highlighting the reliability of these candidate genes. The candidate genes associated with photosynthesis, Chl, LA, and PA were subjected to GO functional enrichment analysis. The results showed that these genes associated with photosynthesis were mainly enriched in GO terms related to threonine-type endopeptidase activity, threonine-type peptidase activity, electron transfer activity, iron ion binding, and other molecular functional category (**Figure 8A**; **Supplementary Table S5**). The candidate genes related to Chl were mainly enriched in GO terms related to xenobiotic transmembrane transporter activity, threonine-type endopeptidase activity, threonine-type peptidase activity, and transferase activity (**Figure 8B**; **Supplementary Table S6**). The candidate genes related to LA were mainly enriched in alpha-(1,2)-fucosyltransferase activity, galactoside 2-alpha-L-fucosyltransferase activity, and fucosyltransferase activity (**Figure 8C**; **Supplementary Table S7**). The candidate genes related to PA were mainly enriched in xenobiotic transmembrane transporter activity, active transmembrane transporter activity, ribonucleotide binding, and carbohydrate derivative binding (**Figure 8D**; **Supplementary Table S8**).

**Figure 9:**
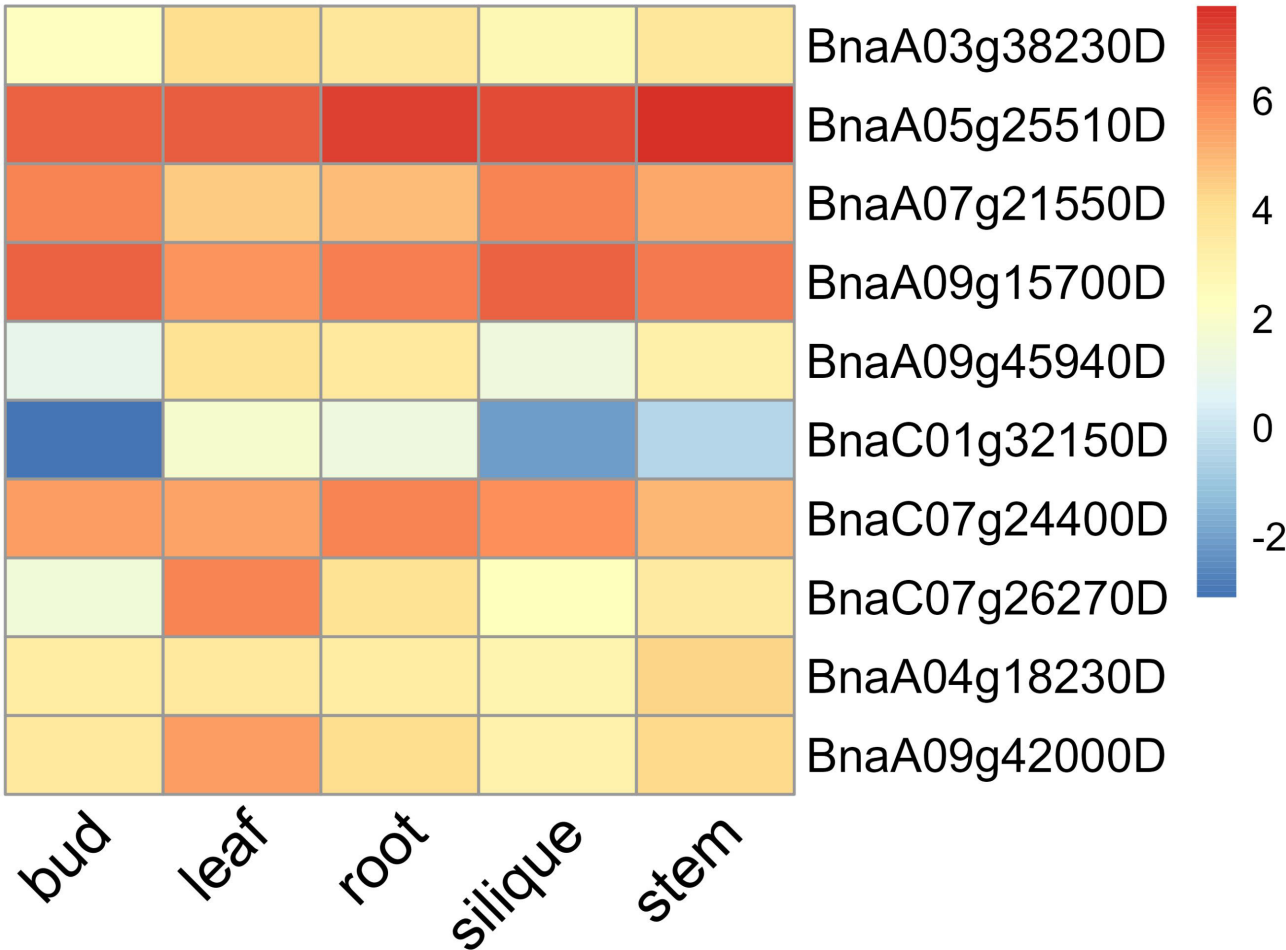
Heatmap of the expression levels of key candidate genes in different tissues (root, stem, leaf, bud, and silique) of rapeseed.

Among the candidate genes associated with photosynthesis, two candidate genes BnaA07g21550D (*NADP-ME4*, *NADP-malic enzyme 4*) and BnaC07g24400D (*CYP709B3*) were found in the stable QTL clusters *q.A7-2* and *q.C7-2*, respectively (**Table 3**). These two candidate genes exhibited high expression levels in leaves and silique walls, and thus might play a critical role in regulating plant growth and photosynthesis (**Figure 9**). Among the candidate genes associated with Chl content, two genes including BnaA09g15700D (*PAF1*, *proteasome alpha subunit F1*) and BnaC01g32150D (*MKK5*, *MAP kinase kinase 5*) were located in the stable QTL clusters *q.A9-2* and *q.C1-6*, respectively (**Table 3**). The expression of BnaA09g15700D was relatively high in all the tested tissues, while the expression of BnaC01g32150D was significantly higher in the leaves than other tissues, indicating these two genes might be the key candidate genes regulating chlorophyll synthesis (**Figure 9**). Among the candidate genes related to LA, two genes [BnaA09g42000D (*NAPRT2, nicotinate phosphoribosyltransferase 2*) and BnaA09g45940D (*WRKY4*, *WRKY DNA-binding protein 4*)] were located in the stable QTL clusters *q.A9-3* and *q.A9-4*, respectively (**Table 3**), which exhibited higher expression levels in the leaves than the other tissues, suggesting that they might be the key genes regulating leaf size (**Figure 9**). Among the candidate genes related to PA, two genes BnaA03g38230D (*CRCK3*, *calmodulin-binding receptor-like cytoplasmic kinase 3*) and BnaA05g25510D (*EIF4A2*, *eukaryotic translation initiation factor 4A2*) were located in the stable QTL clusters *q.A3-8* and *q.A5-4*, respectively (**Table 3**).These genes were highly expressed in leaves and stems, indicating their potential roles in regulating petiole angle (**Figure 9**).

**Table 3:** Key candidate genes associated with photosynthesis and leaf morphology traits identified by GWAS.

QTL cluster	SNP	Trait	Gene id-dormor	Gene id-ZS11	Annotation
*q.A3-8*	A03_19067727	PA, PL	BnaA03g38230D	BnaA03G0389800ZS	calmodulin-binding receptor-like cytoplasmic kinase 3 (CRCK3)
*q.A5-4*	A05_18835949	PA	BnaA05g25510D	BnaA05G0412300ZS	eukaryotic translation initiation factor 4A2 (EIF4A2)
*q.A7-2*	A07_16847439	Ci	BnaA07g21550D	BnaA07G0245100ZS	NADP-malic enzyme 4 (NADP-ME4)
*q.A9-2*	A09_8460857	Chl	BnaA09g15700D	BnaA09G0187300ZS	proteasome alpha subunit F1 (PAF1)
*q.A9-3*	A09_29345053	LW, LA	BnaA09g42000D	BnaA09G0586700ZS	nicotinate phosphoribosyltransferase 2 (NAPRT2)
*q.A9-4*	A09_31652307	LW, LA, PL	BnaA09g45940D	BnaA09G0637400ZS	WRKY DNA-binding protein 4 (WRKY4)
*q.C1-6*	C01_29895643	Chl, PA, LW	BnaC01g32150D	BnaC01G0381800ZS	MAP kinase kinase 5 (MKK5)
*q.C7-2*	C07_31419060	Ci, Gs, Tr, LW	BnaC07g24400D	BnaC07G0275300ZS	cytochrome P450, family 709, subfamily B, polypeptide 3 (CYP709B3)

## Discussion

Photosynthesis is fundamental to plant growth and development, playing a pivotal role in determining crop yields. Therefore, enhancing photosynthetic efficiency is recognized as an effective strategy to increase crop productivity (Kromdijk et al., 2016; Long et al., 2010). Despite its importance, studies investigating the impact of natural genetic variation on photosynthetic efficiency in rapeseed remain limited. In this study, we investigated the gas-exchange parameters of dark reactions in photosynthesis, chlorophyll content, and leaf morphology traits of a natural rapeseed population and identified key loci and genes regulating CO_2_-fixation and associated mechanisms in rapeseed by GWAS.

Our correlation analysis revealed that photosynthesis and chlorophyll content exhibited a significant positive correlation, consistent with previous reports (Gu et al., 2017; Shamim et al., 2022). Interestingly, it has been found that mutants with low leaf Chl content exhibited an increased net photosynthetic rate under the same conditions of light, water, and fertilizer cultivation in barley (Rotasperti et al., 2022). Additionally, we observed a novel significant negative correlation between photosynthesis and leaf area, supporting the hypothesis that lobed leaves may possess a higher photosynthetic capacity. These correlations indicated that optimal light capture and energy conversion might require intricate balance to maintain efficient photosynthesis within limited leaf spatial structure. Our results suggest that other factors such as petiole angle, which determines leaf orientation and petiole length, may play a critical role in photosynthetic efficiency.

Although previous studies have explored the genetic factors affecting the chlorophyll content in rapeseed (Lin et al., 2022; Pang et al., 2022; Ye et al., 2020), limited attention has been given to the genetic mechanisms underlying photosynthesis and leaf morphology traits. In this study, 9 QTL clusters were identified to be associated with photosynthesis, which were co-detected by multiple traits. Additionally, 11 QTL clusters were identified to be associated with leaf morphology, of which 4 QTL clusters (*q.A3-5*, *q.A7-4*, *q.C4-2*, and *q.C7-2*) were co-detected by both photosynthesis traits and leaf morphology traits (**Table 2**). Furthermore, many of the candidate genes identified were associated with both photosynthetic and leaf morphology traits, underscoring the complexity of the genetic architecture governing these traits and the potential pleiotropic effects of these candidate genes. Our findings contribute to the understanding of the genetic control of photosynthesis and leaf morphology in rapeseed. Specifically, 60 QTNs were identified to be associated with chlorophyll content, and mapped to 3 QTL clusters located on chromosomes A4, A9, and C1, respectively. These loci differ from those previously reported for chlorophyll content, which were primarily located on chromosomes A01, A02, and A03 (Lin et al., 2022; Pang et al., 2022; Ye et al., 2020). This indicates that the QTL clusters identified in our study represent novel loci, thereby expanding the known genetic landscape related to chlorophyll content in rapeseed.

The GO enrichment analysis of the candidate genes related to photosynthesis and leaf morphology provides a deeper understanding of the molecular functions and biological processes. Threonine-type endopeptidases may contribute to maintaining photosynthetic efficiency by participating in the turnover of photosynthetic proteins within chloroplasts, facilitating the degradation and recycling of damaged or unnecessary proteins. Our data showed that the genes related to photosynthesis and chlorophyll content were enriched in threonine-type endopeptidase activity pathway (**Figures 8A, B**). Similarly, candidate genes associated with LA were enriched in the fucosyltransferase activity pathway, which plays an important role in the formation of plant cell walls (Cicéron et al., 2016; Oikawa et al., 2013), leaf growth and plant development.

The identification of candidate genes in stable QTL clusters related to photosynthesis traits in rapeseed enhances our understanding of the genetic architecture of these complex phenotypes. The *NADP-ME4* gene is involved in the malate-aspartate shuttle, which helps balance cellular redox states and supplies carbon skeletons for the Calvin cycle (Igamberdiev, 2003). The *NADP-ME* subtype is an effective C_4_ photosynthesis pathway, as it as it modulates the PEPCK activity to optimize light capture, maintaining a high photosynthetic rate under varying light conditions (Bellasio and Lundgren, 2024). Although NADP-ME is primarily involved in malate metabolism, its direct role in the C_3_ photosynthetic pathway is less clear. Nevertheless, the metabolic network within plant cells is highly complex and interconnected, and NADP-ME may indirectly influence the C_3_ photosynthetic pathway by affecting intracellular malate levels and the availability of NADPH. *CYP709B3*, encoding a cytochrome P450 monooxygenases, affects photosynthetic efficiency by modulating electron transport in photosystemII (Larom et al., 2015; Mellor et al., 2017). Our GWAS and gene expression analyses identified two genes, BnaA07g21550D (*NADP-ME4*) and BnaC07g24400D (*CYP709B3*), as key candidate genes involved in photosynthesis and plant growth, particularly in leaves and silique walls.

PAF1 (proteasome alpha subunit F1) is a component of the 26S proteasome, which plays a crucial role in the regulation of protein turnover within cells. The 26S proteasome is involved in various cellular processes such as the degradation of damaged and misfolded proteins (Barman et al., 2024). MKK5 (MAP kinase kinase 5) is an enzyme involved in the mitogen-activated protein kinase (MAPK) signaling pathway, playing a crucial role in regulating various cellular processes such as stress responses, cell growth, differentiation, and apoptosis (Zhang and Zhang, 2022). In addition, our study found that these two genes, BnaA09g15700D (*PAF1*) and BnaC01g32150D (*MKK5*), are located in the QTL region that is significantly associated with Chl content. These two genes exhibit higher expression in leaves compared to other tissues, indicating their roles as key regulators of chlorophyll synthesis. *WRKY4*, a transcription factor, is involved in the regulation of plant growth and development, and the response to biotic and abiotic stresses (Li P. et al., 2021). In this study, BnaA09g45940D (*WRKY4*) and BnaA09g42000D (*NAPRT2*) were identified within stable QTL clusters (*q.A9-3* and *q.A9-4*) associated with leaf area, suggesting that these two genes are key regulators of leaf size and development.

## Conclusion

In summary, this study identified 21 stable QTL clusters and 10 key candidate genes through GWAS, and elucidated their roles in regulating photosynthesis and leaf morphology in rapeseed. These findings deepen our understanding of the genetic architecture underlying photosynthesis and leaf development and provide valuable insights that could facilitate the breeding of high-yield, high-quality rapeseed varieties with enhanced photosynthetic efficiency. This research lays a strong foundation for future genetic improvement efforts in rapeseed cultivation.

## Data Availability

The transcriptome datasets presented in this study can be found in online repositories https://yanglab.hzau.edu.cn/BnIR/expression_zs11. The genotype data is uesed as the 50k SNP chip which has been used in the paper of Qin et al., 2022. The original contributions presented in the study are included in the article/**Supplementary Material**, further inquiries can be directed to the corresponding author.

## Supplementary material

Supplementary Figure 1 Distribution of QTNs associated with photosynthetic and leaf morphological traits on chromosomes. The horizontal axis represents different chromosomes, and the vertical axis indicates different traits. Different colored dots denote QTNs detected in different years. net photosynthetic rate (Pn), leaf chlorophyll content (Chl), transpiration rate (Tr), intercellular carbondioxide concentration (Ci), stomatal conductance (Gs), petiole angle (PA), leaf area (LA), leaf length (LL), leaf width (LW), and petiole length (PL).

Supplementary Figure 2 Phenotypic differences between two genotypes of QTN A04_15190622 significantly associated with different traits. **(A-E)** Box plots of allelic phenotypic variations of QTN A04_15190622 for net photosynthetic rate (Pn), transpiration rate (Tr), intercellular carbondioxide concentration (Ci), stomatal conductance (Gs), and leaf width (LW), respectively. Significant differences between the alleles were assessed using a two-tailed t-test, ** indicates P < 0.01, **** indicates P < 0.0001.

Supplementary Figure 3 Phenotypic differences between two genotypes of QTN A09_325988 significantly associated with different traits. **(A-D)** Box plots of allelic phenotypic variations of QTN A09_325988 for intercellular carbondioxide concentration (Ci), stomatal conductance (Gs), transpiration rate (Tr), and leaf width (LW), respectively. Significant differences between the alleles were assessed using a two-tailed t-test, *** indicates P < 0.001, **** indicates P < 0.0001.

Supplementary Figure 4 Phenotypic differences between two genotypes of QTN A09_29345053 significantly associated with different traits. **(A-F)** Box plots of allelic phenotypic variations of QTN A09_29345053 for transpiration rate (Tr), leaf chlorophyll content (Chl), leaf area (LA), leaf length (LL), leaf width (LW), and petiole length (PL), respectively. Significant differences between the alleles were assessed using a two-tailed t-test, * indicates P < 0.05, ** indicates P < 0.01, *** indicates P < 0.001, **** indicates P < 0.0001.

Supplementary Table 1 Detailed information on trait-related significant QTNs identified by GWAS.

Supplementary Table 2 Detailed information on significant QTNs repeated identified by GWAS.

Supplementary Table 3 Detailed information on key trait-related QTNs identified by GWAS.

Supplementary Table 4 List of candidate genes associated with photosynthetic and leaf morphological traits.

Supplementary Table 5 GO enrichment results of candidate genes associated with photosynthetic traits.

Supplementary Table 6 GO enrichment results of candidate genes associated with chlorophyll content.

Supplementary Table 7 GO enrichment results of candidate genes associated with leaf area.

Supplementary Table 8 GO enrichment results of candidate genes associated with petiole angle.
