# Supplementary figures and images for: Identification of genetic loci and candidate genes regulating photosynthesis and leaf morphology through genome-wide association study in Brassica napus L
Source: Front Plant Sci. 2024 Dec 20;15:1467927. doi: 10.3389/fpls.2024.1467927 (PMC11695134; doi:10.3389/fpls.2024.1467927)

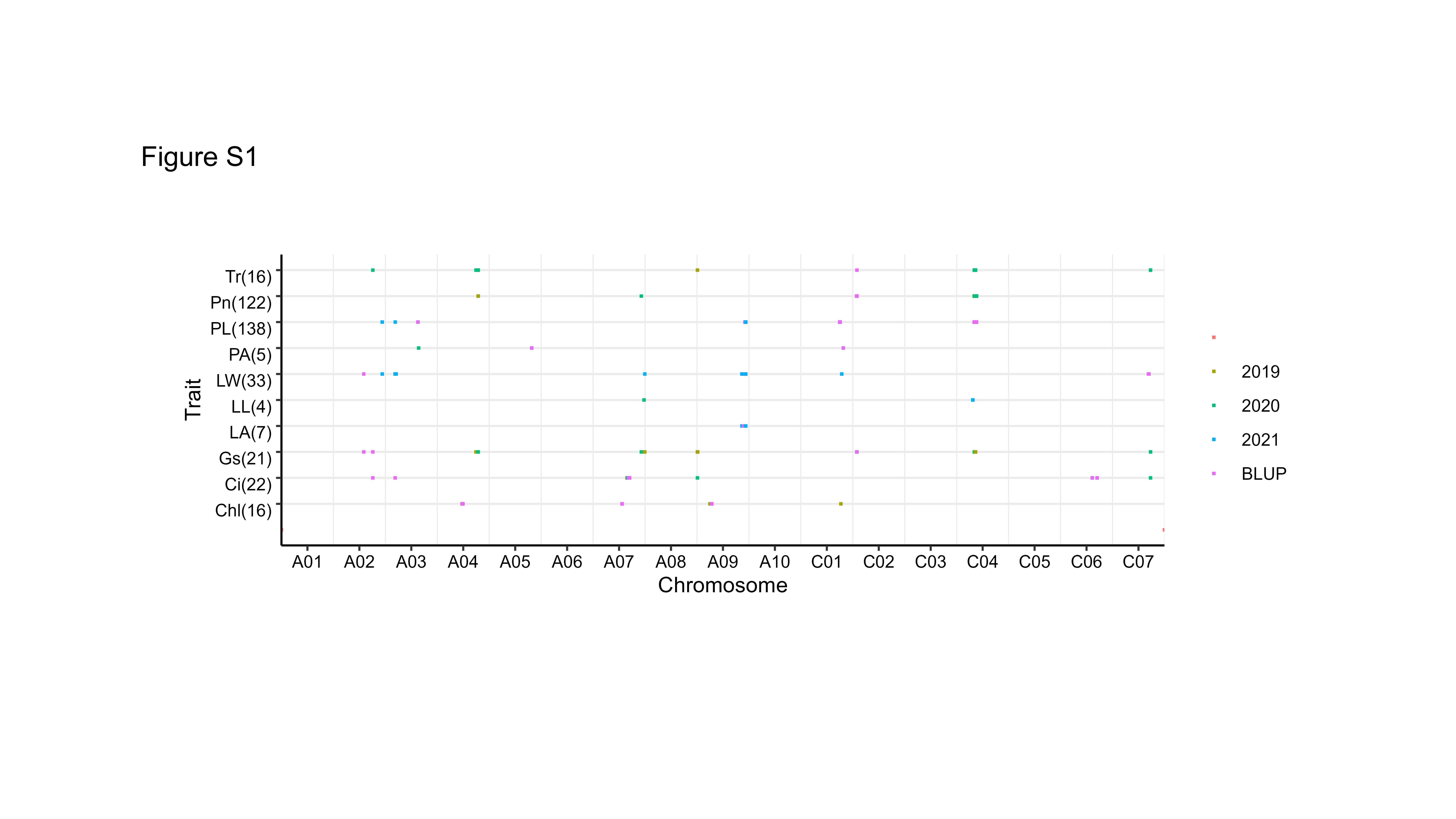

Supplement: Supplementary Figure 1 — Distribution of QTNs associated with photosynthetic and leaf morphological traits on chromosomes. The horizontal axis represents different chromosomes, and the vertical axis indicates different traits. Different colored dots denote QTNs detected in different years. net photosynthetic rate (Pn), leaf chlorophyll content (Chl), transpiration rate (Tr), intercellular carbondioxide concentration (Ci), stomatal conductance (Gs), petiole angle (PA), leaf area (LA), leaf length (LL), leaf width (LW), and petiole length (PL). [file Image1.jpeg]

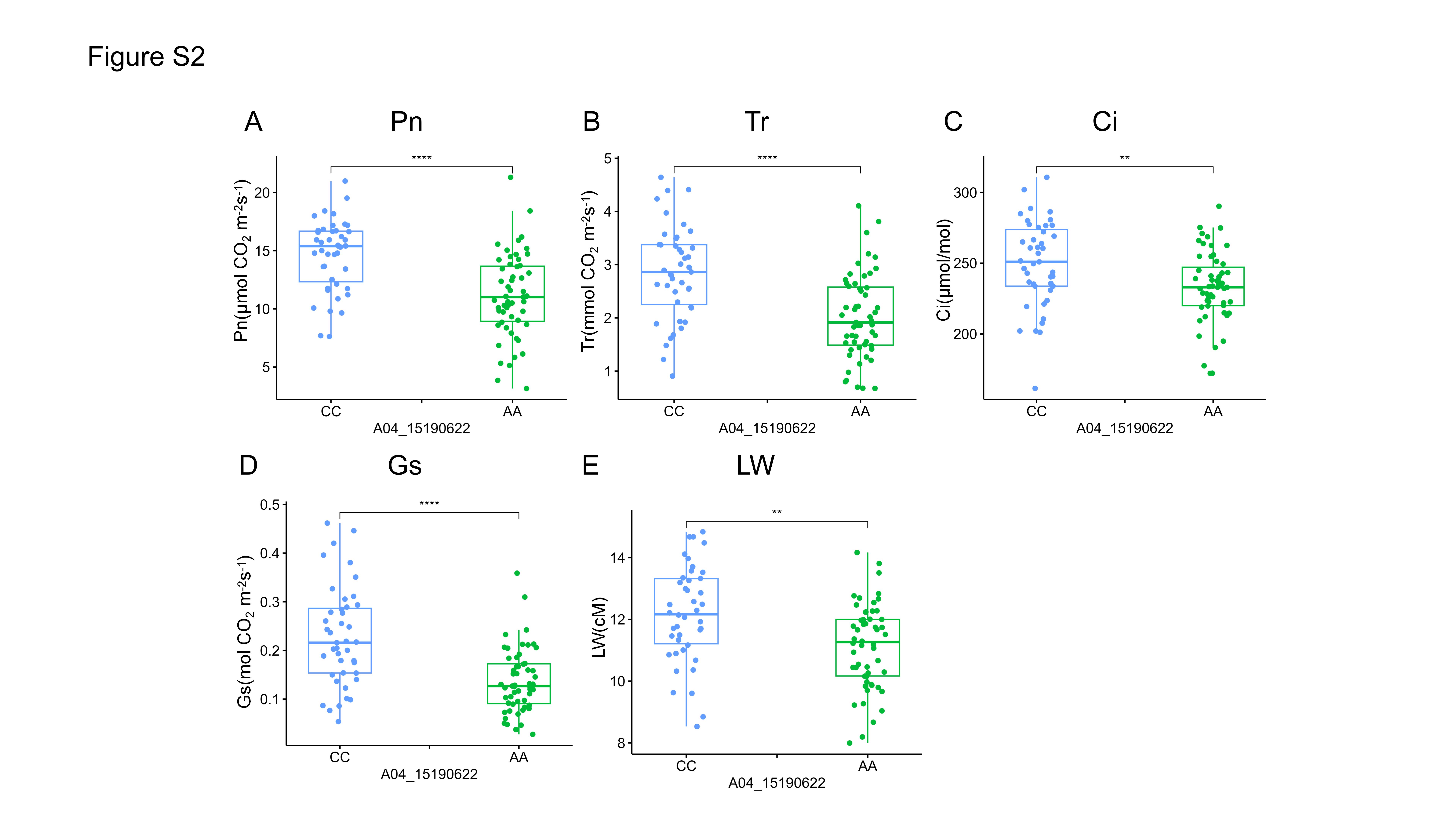

Supplement: Supplementary Figure 2 — Phenotypic differences between two genotypes of QTN A04_15190622 significantly associated with different traits. (A-E) Box plots of allelic phenotypic variations of QTN A04_15190622 for net photosynthetic rate (Pn), transpiration rate (Tr), intercellular carbondioxide concentration (Ci), stomatal conductance (Gs), and leaf width (LW), respectively. Significant differences between the alleles were assessed using a two-tailed t-test, ** indicates P < 0.01, **** indicates P < 0.0001. [file Image2.jpeg]

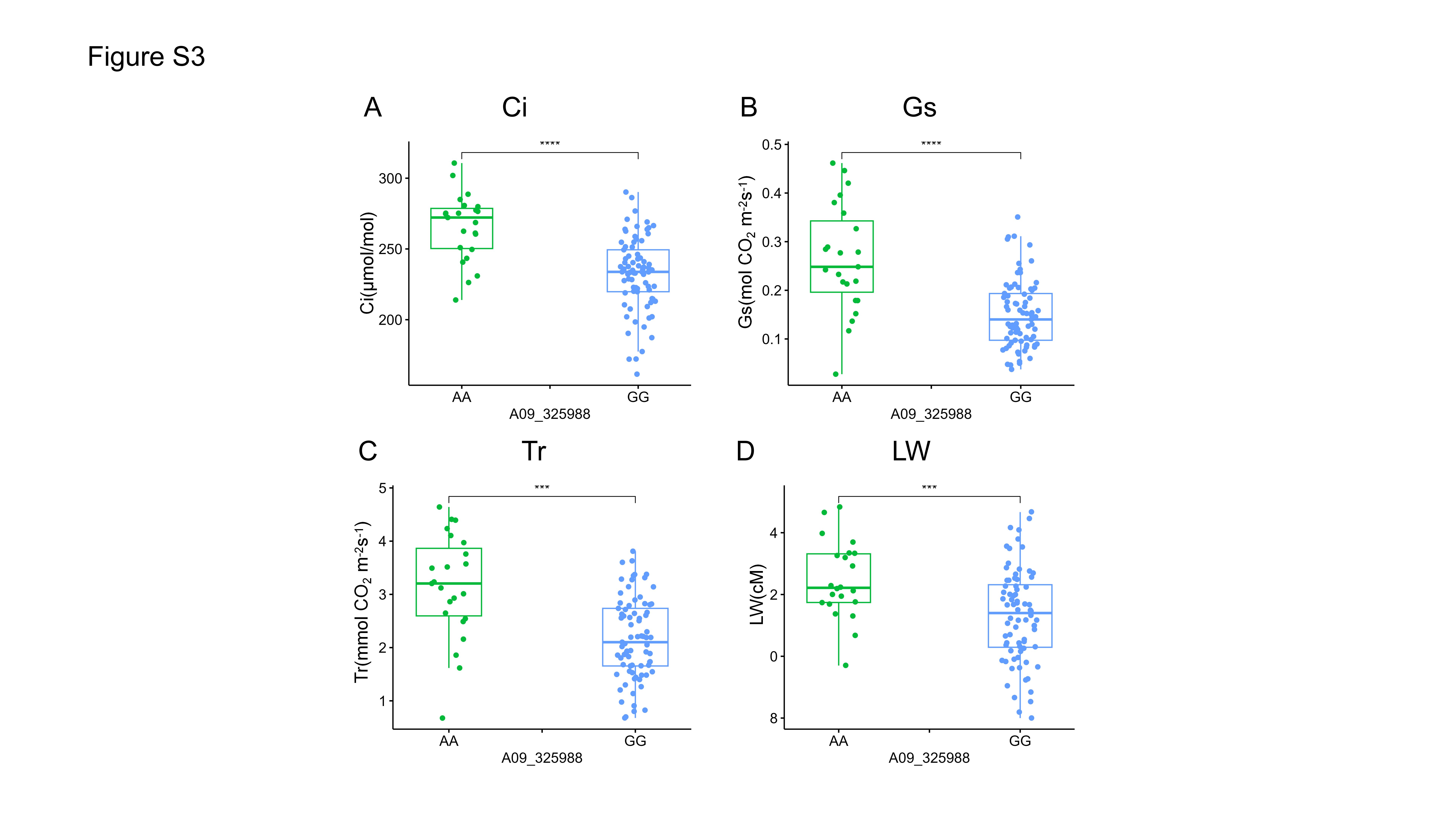

Supplement: Supplementary Figure 3 — Phenotypic differences between two genotypes of QTN A09_325988 significantly associated with different traits. (A-D) Box plots of allelic phenotypic variations of QTN A09_325988 for intercellular carbondioxide concentration (Ci), stomatal conductance (Gs), transpiration rate (Tr), and leaf width (LW), respectively. Significant differences between the alleles were assessed using a two-tailed t-test, *** indicates P < 0.001, **** indicates P < 0.0001. [file Image3.jpeg]

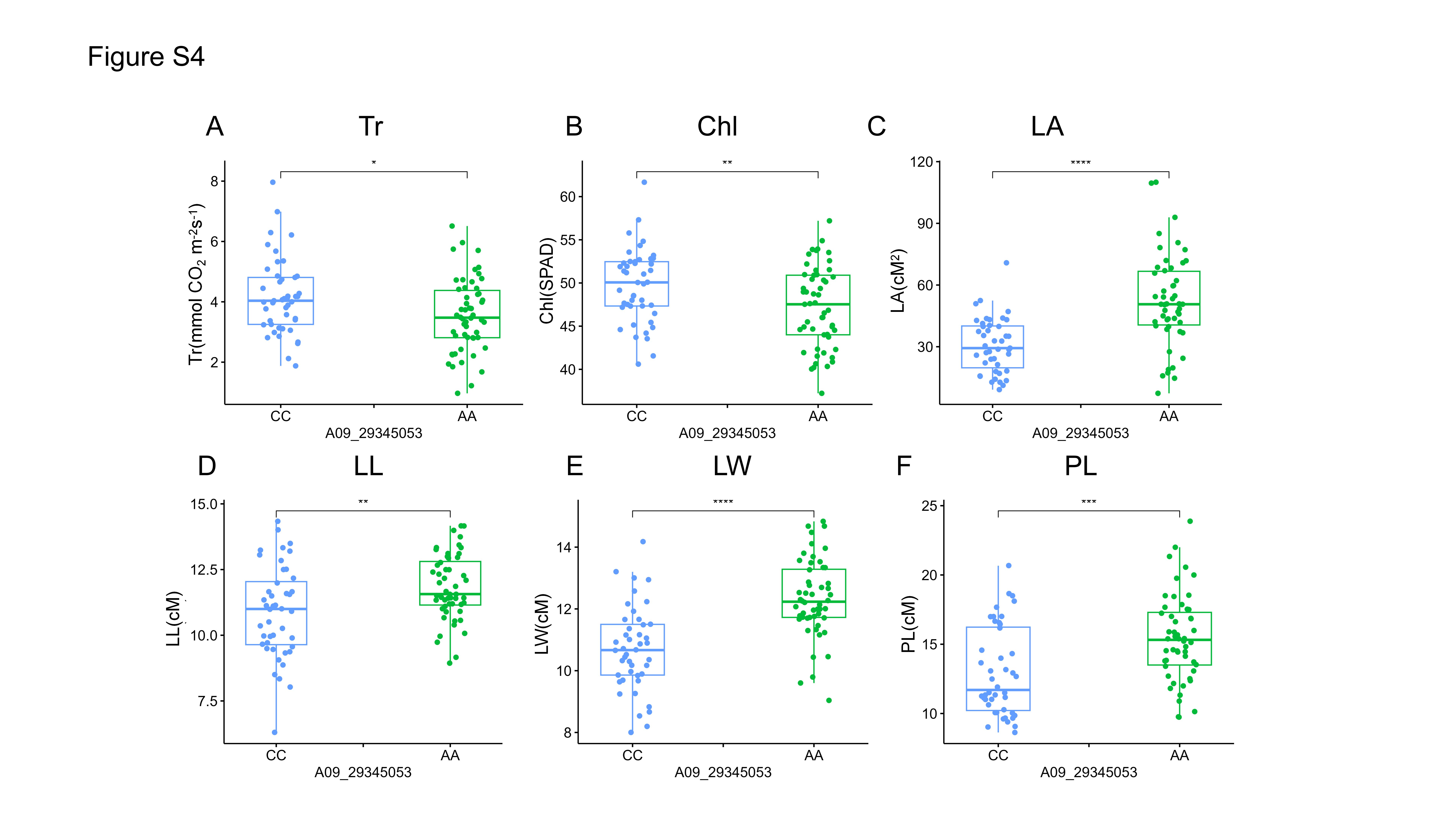

Supplement: Supplementary Figure 4 — Phenotypic differences between two genotypes of QTN A09_29345053 significantly associated with different traits. (A-F) Box plots of allelic phenotypic variations of QTN A09_29345053 for transpiration rate (Tr), leaf chlorophyll content (Chl), leaf area (LA), leaf length (LL), leaf width (LW), and petiole length (PL), respectively. Significant differences between the alleles were assessed using a two-tailed t-test, * indicates P < 0.05, ** indicates P < 0.01, *** indicates P < 0.001, **** indicates P < 0.0001. [file Image4.jpeg]
